# Convergent Functional Genomic Evolution Underlying Repeated Freshwater Colonization in Cetaceans

**DOI:** 10.1007/s00239-026-10325-4

**Published:** 2026-06-22

**Authors:** D. Pinilla-Beltrán, Leonardo Duarte-Santos, Mariana F. Nery

**Affiliations:** Laboratory of Evolutionary Genomics, Department of Genetics, Evolution, Immunology and Microbiology, University of Campinas, Cidade Universitária, Campinas, Sao Paulo 13083-862 Brazil

**Keywords:** Freshwater adaptation, Cetacean evolution, Comparative genomics, Positive selection, Convergent evolution

## Abstract

The transition from marine to freshwater environments represents a remarkable evolutionary shift in cetaceans, yet the genomic underpinnings of this adaptation remain poorly understood. This study investigates the genomic signatures of freshwater adaptation in riverine cetaceans through comparative evolutionary analyses of six species: *Inia geoffrensis*, *Lipotes vexillifer, Platanista gangetica*, *Platanista minor*, *Neophocaena asiaeorientalis asiaeorientalis*, and *Sotalia fluviatilis*. By integrating positive selection analysis, gene family dynamics, and evolutionary rate convergence, we identified key molecular adaptations associated with freshwater colonization. The analysis revealed that five of the six species exhibited positive selection in the NSMAF and CTRL genes, suggesting widespread selective pressures related to inflammatory responses and digestive adaptations, respectively. Functional enrichment analyses revealed adaptive signatures in hematopoiesis, osmoregulation, skeletal development, and immune responses, reflecting the physiological challenges of freshwater environments. Gene family evolution analyses using CAFE identified dynamic patterns of expansions and contractions in immune-related genes, transcriptional regulation, and cell adhesion pathways across riverine lineages. Relative evolutionary rate (RER) analysis using RERconverge identified 95 genes showing convergent rate shifts associated with freshwater adaptation, including genes involved in cellular nitrogen compound responses and transcriptional regulation. Despite positively selected genes overlap was limited and did not follow simple clade-wide patterns, our results demonstrate that freshwater adaptation in cetaceans involves putative convergent evolution of fundamental biological systems, including immune responses, metabolic regulation, and morphological development. These findings provide new insights into the molecular mechanisms underlying speciation in aquatic mammals and highlight critical biological pathways that have enabled the successful colonization of freshwater ecosystems by multiple independent cetacean lineages.

**Supplementary Information:**

The online version contains supplementary material available at 10.1007/s00239-026-10325-4.

## Introduction

The transition from terrestrial to aquatic environments represents one of the most remarkable macroevolutionary shifts in mammalian history. Cetaceans exemplify this transition, having evolved from terrestrial artiodactyl ancestors to fully aquatic marine species during the Eocene (~ 50 Mya) (Thewissen and Williams 2002; Gingerich 2008; Kelley and Pyenson 2015). Although this marine adaptation has been intensively studied, a secondary transition, less investigated, occurred when multiple odontocete lineages independently colonized freshwater systems.

The so-called “river dolphins” constitute the most emblematic of these reinvasions. The four classical genera (*Inia*, *Pontoporia*, *Platanista*, and the functionally extinct *Lipotes*) represent phylogenetically distant odontocete lineages that independently derived from marine ancestors and converged on a similar riverine ecological niche (Reeves and Martin 2009; Smith and Reeves 2012; Kelley and Pyenson 2015). However, whereas *Inia*, *Platanista,* and *Lipotes* inhabit riverine or freshwater systems, *Pontoporia blainvillei* is currently restricted to shallow coastal and estuarine marine waters of the southwestern Atlantic (Hamilton et al. 2001). For this reason, in this study, we focused on extant lineages that are obligate riverine or predominantly freshwater and did not include *Pontoporia* as part of the freshwater focal set. In addition to this core group, other freshwater cetaceans, such as *Sotalia fluviatilis*, *Orcaella brevirostris*, and *Neophocaena asiaeorientalis*, inhabit riverine or estuarine environments but derive from more recent colonization events involving coastal marine sister taxa (Beasley et al. 2005; Caballero et al. 2008). Together, all these independent invasions provide a natural replicated framework to investigate how distinct evolutionary histories can yield similar adaptive outcomes under comparable environmental pressures.

Riverine habitats impose a set of ecological challenges, including navigation through shallow and structurally complex waterways, foraging under conditions of chronically reduced visibility, and the need for short-range echolocation in highly turbid waters (Hamilton et al. 2001; de Muizon et al. 2018). These conditions have driven notable morphological convergence among riverine cetaceans, particularly in cranial architecture. Page and Cooper (2017) demonstrated significant convergence in cranial shape across phylogenetically distant river dolphin species, characterized by elongation of rostral and associated feeding structures that facilitate piscivorous foraging. Similarly, do Val et al. (2025) reported morphological similarities among Amazonian dolphin species related to both feeding mechanics and spatial navigation.

Convergent adaptive responses are also reflected at the molecular level. Evidence of positive selection and convergent evolution has been documented in mitochondrial genes, including *ND2* (Caballero et al. 2008, 2015), in osmoregulatory pathways such as *AVP*, *AQP2*, and *SLC14A2* (Pedro et al. 2015; Shen et al. 2012; Zhou et al. 2018; Ramos et al. 2023), and in echolocation-related sensory genes (Magpali et al. 2024). Also, immunological adaptations in the TLR4 gene were associated with freshwater pathogen resistance (Shen et al. 2012). These findings indicate that similar selective pressures may promote repeated molecular solutions associated with freshwater specialization. However, most genomic studies to date have focused on a few riverine lineages, limiting our understanding of whether shared adaptive signatures are widespread and repeatable across independent freshwater invasions.

Changes in gene family composition, through expansion, contraction, and functional turnover, represent another mechanism contributing to adaptive divergence in mammals (Demuth and Hahn 2009). In cetaceans, such processes have been linked to traits including oxidative stress response, thermogenesis, and sensory function (Zhou et al. 2019; Sun, et al. 2017; Tian et al. 2019; Yuan et al. 2021). Yet, no study has systematically evaluated whether gene family dynamics also exhibit convergent patterns among freshwater taxa, despite repeated transitions into similar ecological contexts.

Given the complex evolutionary history of freshwater colonization in odontocetes (toothed whales)—one of the two extant cetacean suborders alongside mysticetes (baleen whales)—uncovering the genomic mechanisms underlying riverine adaptation is important to understanding how independent lineages respond to similar ecological challenges. Freshwater cetaceans represent multiple transitions from marine to riverine environments, providing a natural system of evolutionary replication for testing genomic convergence.

Accordingly, here we investigated genomic signatures of adaptation and convergence across freshwater cetaceans using a comparative framework that includes all extant obligate river dolphin lineages (*Inia geoffrensis, Lipotes vexillifer,* and *Platanista* spp.), as well as species derived from more recent freshwater colonization events (*Sotalia fluviatilis* and *Neophocaena asiaeorientalis*). Specifically, we (i) identified positively selected genes to detect molecular signatures associated with riverine adaptation, (ii) evaluated gene family expansions and contractions to assess convergent patterns potentially linked to freshwater ecological pressures, and (iii) performed evolutionary rate convergence analyses to quantify the degree of molecular convergence among independent freshwater lineages.

## Material and Methods

### Genomic Data

We analyzed whole-genome sequences from 20 species, including 19 cetaceans and the outgroup *Hippopotamus amphibius*, as the hippopotamus is the closest extant relative of cetaceans within Artiodactyla*.* The freshwater focal set comprised six odontocete lineages (*Inia geoffrensis*, *Platanista gangetica*, *Platanista minor*, *Lipotes vexillifer*, *Sotalia fluviatilis*, and *Neophocaena asiaeorientalis*). The remaining 13 species correspond to marine odontocetes and mysticetes, selected to provide broad phylogenetic and ecological coverage of marine cetacean diversity while relying on high‑quality genome assemblies, as detailed in Supplementary file 1, Table S1. All genome assemblies were retrieved from NCBI, and accession numbers are provided in the same Supplementary Table 1. As briefly explained before, we did not include *Pontoporia blainvillei* as a freshwater species because, despite its historical classification among the ‘river dolphins’ based on phylogeny and morphology (Hamilton et al. 2001), the extant *P. blainvillei* inhabits shallow coastal and estuarine marine waters rather than river channels. Since our objective was to isolate genomic signatures of adaptation to freshwater environments, including a marine relative would have introduced confounding variables.

Unless otherwise stated, all downstream analyses were conducted using the same species tree topology. This topology was used consistently for positive selection and relative evolutionary rate analyses to ensure comparability across methods. The scripts and code used to perform the comparative genomic, positive selection, gene family, and evolutionary rate analyses are publicly available at: https://github.com/Danipinibel/cetacean_genomics_pipeline_structure.git

### Gene Annotation and Orthology Inference

Protein-coding genes for *I. geoffrensis*, *P. gangetica*, *P. minor*, and *S. fluviatilis* were annotated using a multi-step pipeline. First, GeMoMa v1.9 (Keilwagen et al. 2016) was run with *Tursiops truncatus, Delphinus delphis*, and *Orcaella brevirostris* as references, using the GAF module and applying a stringent iAA ≥ 0.9 filter. Additional gene prediction was incorporated using Braker3 (Bruna et al. 2023) with protein evidence from *T. truncatus* and *D. delphis*, and Tiberius (Gabriel et al. 2024) for ab initio predictions. Finally, the annotations generated by GeMoMa, Braker3, and Tiberius were integrated into a final consensus set using OMAannotator (Altenhoff et al. 2019). Annotation completeness was evaluated with BUSCO (Simão et al. 2015) using the mammalia_odb10 dataset (Supplementary file 1: Table S2). Species lacking existing gene annotations were annotated in this study, whereas species with available annotations were used as provided. Coding sequences (CDS) were extracted with gffread (https://github.com/gpertea/gffread).

We identified orthologous gene groups for all species using the OrthoFinder v2.5.2,b after selecting the longest transcript per gene with all parameters set to default. Single-copy orthologs were defined as orthogroups containing exactly one gene copy per species, resulting in a final set of 9776 single-copy orthologs. Restricting downstream analyses to single-copy orthologs minimizes potential biases associated with differences in genome assembly or annotation quality, such as gene fragmentation, redundancy, or lineage-specific annotation artifacts. Multiple sequence alignments of each single-copy ortholog were generated at the codon level with MACSE 2.03 (Ranwez et al. 2011), which accounts for frameshifts and stop codons.

### Positive Selection Analyses

We performed branch-site tests to detect positive selection acting on specific sites and lineages (Álvarez-Carretero et al. 2023; Yang and Nielsen 2002). Separate tests were conducted for each freshwater cetacean lineage (*I. geoffrensis, L. vexillifer, P. minor, P. gangetica, S. fluviatilis, and N. asiaeorientalis*), with the focal lineage specified as the foreground branch. Additionally, we performed an analysis in which all freshwater lineages were jointly specified as foreground branches to test for signals of positive selection shared across riverine taxa. We opted to use branch-site models because branch models average substitution rates over branches and sites and thus may fail to detect diversifying selection when it affects only a subset of sites (Yang 2007; Yang and Nielsen 2002). These tests are based on the estimation of ω = dN/dS, which measures the ratio between the nonsynonymous (dN) and synonymous (dS) substitution rates. Neutral selection is inferred when ω = 1, whereas ω < 1 and ω > 1 indicate purifying and positive selection, respectively (Yang 2007).

In each analysis, the focal species was set as the foreground branch (with potential ω > 1), while all other branches formed the background. The alternative model (Model A) allowed a class of sites to evolve under positive selection (ω_2_ > 1) in the foreground branch, whereas the null model constrained these sites to neutrality (ω_2_ = 1). Likelihood ratio tests (LRTs) were conducted between these models, with χ^2^-derived p-values (df = 1; significance threshold p < 0.05). Sites under positive selection were identified using Bayesian Empirical Bayes (BEB) posterior probabilities ≥ 0.95 (Yang et al. 2005; Yang 2007). All analyses were implemented in codeml (PAML v4.10.7j) under default parameters, with the species tree topology fixed for consistency across runs. To account for multiple testing across genes and lineages, all LRT p-values were corrected using a false discovery rate (FDR) approach following the Benjamini–Hochberg procedure, and only FDR-adjusted p-values < 0.05 were considered significant. For downstream comparisons, we evaluated the presence of positively selected genes across pairs, trios, quartets, quintets, and all six freshwater species to identify genes shared among multiple lineages.

To test whether PSG overlap among freshwater cetaceans was associated with phylogenetic relatedness or shared river habitats, we analyzed pairwise species combinations. Each pair was classified as phylogenetically close or distant, and as sharing the same river basin or not. We compared the number of PSGs shared between groups using Welch’s t-tests and evaluated the joint effects of phylogeny and habitat using a two-factor ANOVA. We also tested for regional differences in PSG overlap between Asian and American lineages. Statistical significance was assessed at p < 0.05.

### Gene Family Evolution

To detect gene family expansions and contractions associated with freshwater adaptation, we conducted a CAFE v4.2 analysis (Mendes et al. 2020) using the ultraconserved elements (UCE) phylogeny as the species tree. In this analysis, *Hippopotamus amphibius* was included as an outgroup to appropriately root gene family size evolution within Cetacea. UCE loci were identified and analyzed through the Phyluce pipeline (Faircloth 2016). To prepare the genomic data, raw reads were processed using Phyluce’s illumiprocessor, which integrates Trimmomatic (Bolger et al. 2014) with default quality filtering and adapter trimming settings as implemented in the Phyluce pipeline. UCE loci were identified from the assembled contigs using a UCE probe sequence file downloaded from the repository (Faircloth 2016; https://github.com/faircloth-lab/uce-probe-sets/tree/master/uce-5k-probe-set). Contigs containing UCE loci were extracted, and duplicate sequences were removed to avoid redundancy. Alignment of contig sequences with UCE probe sequences was performed using Lastz (Harris 2007), a tool optimized for pairwise genomic alignments. Multiple sequence alignments of UCE loci were subsequently refined using the EDGE trimming method implemented in MAFFT (Katoh and Standley 2013) to ensure precise and high-quality alignments. The best-fit model of sequence evolution was determined using ModelFinder (Kalyaanamoorthy et al. 2017), implemented in IQ-TREE version 1.6-8 (Nguyen et al. 2015).

Divergence times were estimated using BEAST v2.3.6 through the CIPRES Science Gateway (Miller et al. 2010). Analyses used a relaxed lognormal molecular clock under the calibrated Yule speciation process. The GTR substitution model with empirically estimated base frequencies was applied, as it was the best model selected by ModelFinder in IQ-TREE. A single Markov Chain Monte Carlo (MCMC) runs were conducted for 100 million generations, sampling every 10,000 generations. Posterior distributions of parameters were assessed using Tracer v.1.7.1 (Strimmer and Rambaut 2002), and trees were summarized using TREEANNOTATOR (Bouckaert et al. 2014) with a 10% burn-in. We used calibration points derived from previously published studies (Xiong et al. 2009; McGowen et al. 2020) (Supplementary file 1: Table 3).

CAFE analyses were then performed using the six freshwater cetacean lineages as focal branches, with λ estimated from the data (− s option) and all other parameters left at their default settings. Gene families showing significant expansion or contraction were identified as those exhibiting a statistically significant increase or decrease in family size along a focal branch relative to expectations under the fitted global birth–death model, using a stringent significance threshold (p < 0.01).

### Convergent Evolutionary Rates

To identify genes exhibiting evolutionary rate changes associated with freshwater adaptation, we performed a relative evolutionary rate (RER) analysis using the RERconverge R package version 1.01 (Kowalczyk et al. 2019). RERconverge rapidly computes the association between gene-specific RER and the user-specified traits for large sets of genes. The program estimates the correlation between gene tree branch lengths and the values for traits along those branches using (by default) Kendall’s Tau for binary traits (Kowalczyk et al. 2019). The method computes relative evolutionary rates (RERs) that reflect whether a given gene is evolving slower or faster than the genome-wide average along a branch of the phylogeny (Redlich et al. 2024).

To run RERconverge, we used only single-copy orthologs from Orthofinder. Branch lengths for each gene were estimated using the R package phangorn (Schliep 2011) with codon alignments and the species tree topology generated by Orthofinder as input. Gene trees were used as input for the construction of the master tree and for the estimation of relative evolutionary rates (RERs). Subsequently, a binary trait analysis was performed to associate RERs with habitat type (freshwater vs. marine) using a trait tree where freshwater cetaceans were labeled as foreground (branch length = 1) and all other species were labeled as background (branch length = 0). The correlateWithBinaryPhenotype function was then applied using default parameters to test for an association between RERs and freshwater habitat occupation across the phylogeny, using Kendall’s rank correlation coefficient. As no significant results were obtained after multiple-testing correction using the Benjamini–Hochberg procedure implemented in RERconverge, we adopted an unadjusted significance threshold of p < 0.01 for downstream analyses.

### Gene Ontology and Pathway Enrichment Analyses

To characterize the functional context of genes associated with freshwater adaptation, Gene Ontology (GO) analyses were performed separately for each evolutionary signal detected. Genes identified as being under positive selection and genes exhibiting convergent evolutionary rate shifts in the RERconverge analysis were subjected to functional enrichment analyses using Metascape (http://metascape.org). For these analyses, gene lists were tested against a background set comprising all single-copy orthologs, and enrichment was evaluated across GO Biological Processes and KEGG pathways. Statistical significance was assessed using hypergeometric tests, and multiple testing correction was applied using the Benjamini–Hochberg false discovery rate (FDR), with adjusted p-values < 0.05 considered significant.

In contrast, for gene families identified as significantly expanded or contracted in the CAFE analysis, functional characterization was performed through Gene Ontology annotation using the UniProt database. These annotations were used for descriptive interpretation of gene family functions rather than for formal statistical enrichment testing.

For the GO enrichment analysis comparing RERconverge and positively selected genes (PSGs), PSGs present in at least three of the six analyzed species were selected. These genes were intersected with genes showing significant rate shifts identified by RERconverge to define the test set for enrichment analyses. This procedure established a combined, biologically relevant background for assessing functional enrichment across both datasets.

## Results

To investigate whether independent transitions from marine to freshwater environments in cetaceans have left detectable and potentially convergent genomic signatures, we integrated three complementary comparative approaches: lineage-specific tests of positive selection, analyses of gene family expansions and contractions, and relative evolutionary rate convergence. Together, these analyses were designed to identify both gene-level and functional patterns associated with freshwater adaptation across multiple independent riverine lineages.

### Patterns of Positive Selection and Gene-Level Convergence

In this study, we interpret the overlap of positively selected genes across freshwater species as putative convergence. While the branch-site framework detects lineage-specific signatures of positive selection, it does not formally demonstrate independent convergent selection events at specific sites. Therefore, the term convergence throughout this section refers to recurrent lineage-specific signals compatible with convergent adaptation rather than statistically demonstrated molecular convergence.

**Fig. 1:**
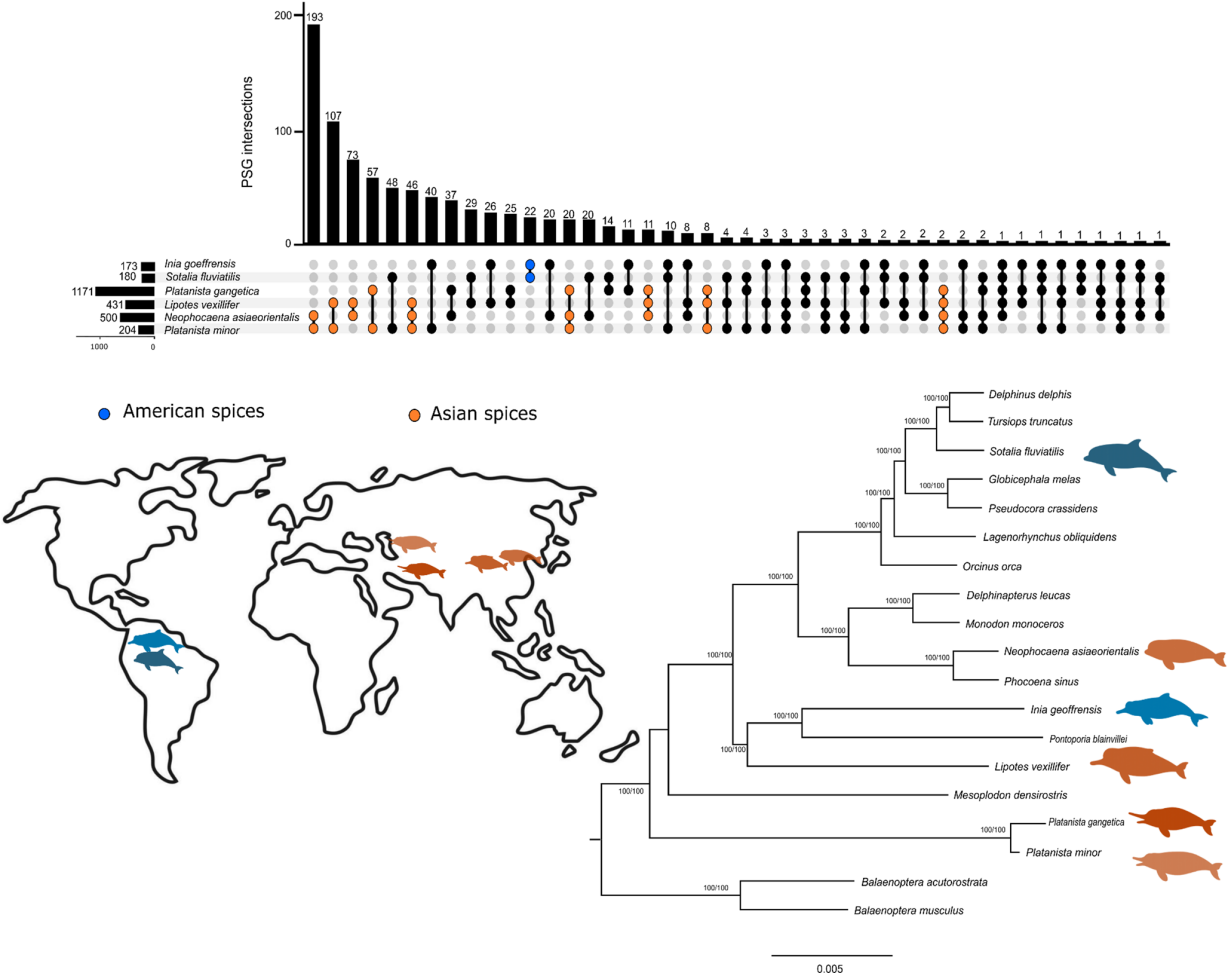
Upset plot illustrating the distribution of positively selected genes across riverine lineages and their overlap patterns. The plot shows the number of genes under positive selection shared among different combinations of species. Blue dots represent South American species, whereas orange dots represent Asian species. At the bottom left, a geographic map shows the species distribution of the riverine taxa. At the bottom right, a maximum likelihood phylogenetic tree depicts the evolutionary relationships among cetaceans, including the riverine lineages (Color figure online)

To identify individual genes that may have undergone rapid adaptive evolution in response to the challenges of freshwater environments, we first performed a genome-wide scan for genes under positive selection (PSGs) across the six riverine lineages. Branch-site analyses identified widespread signatures of positive selection in all six freshwater cetaceans. We identified 1,171 genes showing signatures of positive selection in *Platanista gangetica*, 204 in *Platanista minor*, 500 in *Neophocaena asiaeorientalis*, 431 in *Lipotes vexillifer*, 176 in *Inia geoffrensis*, and 180 in *Sotalia fluviatilis* (Supplementary file 2). Comparative analyses revealed that the overlap of positively selected genes among freshwater species was limited, with no genes shared across all lineages (Fig. 1). Based on these findings, we performed comparative analyses to assess potential signals of adaptive convergence across species, considering whether patterns of convergence are associated with species that are closely related (phylogenetic relatedness) or that inhabit geographically proximate regions, to determine whether adaptive signals reflect shared ancestry or similar environmental conditions. Although no consistent convergence was observed across all species, the following sections further explore recurrent patterns that emerged within specific species combinations, suggesting the influence of both evolutionary history and environmental conditions on adaptive responses to freshwater habitats.

Pairwise comparisons suggested localized structure in the overlap of positively selected genes (PSGs). For example, the Asian river species *Lipotes vexillifer* and *Neophocaena asiaeorientalis* (both from the Yangtze River) and the South Asian taxa *Platanista gangetica* and *Platanista minor* exhibited higher PSG overlap than the American pair *Inia geoffrensis* and *Sotalia fluviatilis* from the Amazon basin (Fig. 1). However, when these patterns were tested statistically using a two-factor ANOVA, neither phylogenetic relatedness nor shared river habitat alone showed a significant effect on PSG overlap (p = 0.764 and p = 0.812, respectively) (Supplementary Fig. S2). In contrast, their interaction was significant (p = 0.027), indicating that adaptive convergence is strongest among species that are both closely related and occupy similar freshwater environments. This suggests that shared ancestry and ecological conditions jointly influence patterns of positive selection.

Notably, a broader comparison revealed that Asian freshwater cetaceans share significantly more PSGs on average than American lineages (Welch’s t-test, p = 0.044) (Supplementary Fig. S2). Together, these results indicate that adaptive convergence reflects both the interaction between phylogeny and environment and an additional regional signal among Asian riverine species.

Comparisons involving three or more species revealed similar structure (Fig. 1). Trios composed exclusively of Asian lineages, such as *Lipotes vexillifer, Platanista gangetica*, and *Neophocaena asiaeorientalis*, shared the highest numbers of PSGs (up to 46). Likewise, the only four-species cluster with shared PSGs (SH2D6 and PARP10) also involved Asian taxa (*P. gangetica, P. minor, L. vexillifer,* and *N. asiaeorientalis*) (Fig. 1c).

Two genes were identified as positively selected in five out of the six species analyzed. NSMAF showed signatures of selection in *I. geoffrensis, L. vexillifer*, *N. asiaeorientalis*, *S. fluviatilis*, and *P. minor*, with positively selected sites located in different regions of the gene across species. Only a single site is shared between *I. geoffrensis* and *S. fluviatilis*, corresponding to a Methionine (M) at alignment position 1472. CTRL was under selection in *P. gangetica, I. geoffrensis, L. vexillifer*, *N. asiaeorientalis,* and *S. fluviatilis* (Fig. 1); however, no positively selected sites are shared across species for this gene. These represent the most broadly shared positively selected genes across taxa and may reflect widespread selective pressures. Notably, the combinations of species exhibiting selection in these genes include both phylogenetically distant and geographically separated taxa, suggesting that these signals cannot be attributed solely to shared ancestry or geographic proximity.

In addition to lineage-specific analyses, we performed a complementary branch-site test considering all six freshwater lineages simultaneously as foreground branches. This analysis identified a total of 2450 genes showing signatures of positive selection (Supplementary file 2). This higher number of positively selected genes is consistent with the substantial number of PSGs detected in each lineage-specific analysis. To assess the relationship between both approaches, we compared the set of PSGs identified in the combined analysis with those detected in individual lineages. We found that 2200 genes (89.8%) were shared between both approaches, whereas 250 genes (10.2%) were uniquely detected in the combined analysis. On average, 69.6% of lineage-specific PSGs were also recovered in the combined test, although this proportion varied across species (range: 59.3–76.1%). These results suggest that while a considerable proportion of adaptive signals is consistently detected across analytical frameworks, the combined analysis also captures additional signals that may reflect selection acting across multiple lineages or increased statistical power when lineages are analyzed jointly.

To determine if the observed molecular changes across different lineages converge on specific physiological or metabolic pathways, we conducted functional enrichment analyses. Functional enrichment analyses based on these positively selected genes should be interpreted within the same framework of putative convergence described above, as they derive from recurrent lineage-specific signals identified by the branch-site analyses.

**Fig. 2:**
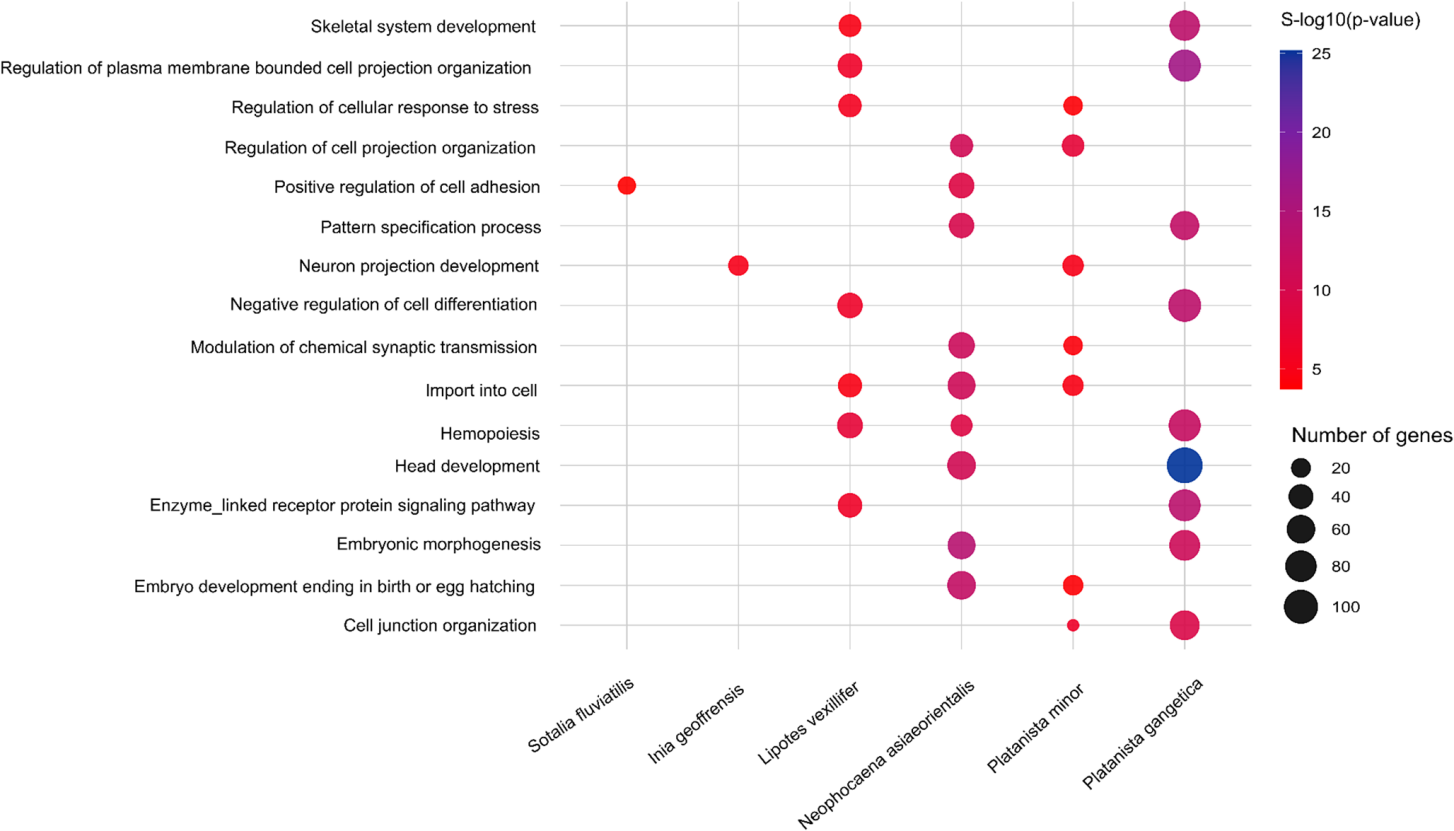
Gene ontology (GO) enrichment analysis of biological processes shared across cetacean species. The y-axis displays GO terms corresponding to biological processes enriched in multiple species. The x-axis represents the analyzed riverine cetacean species. Each dot indicates the enrichment of a GO term in a given species. Dot size reflects the number of genes associated with the GO term, and color intensity indicates statistical significance, represented as –log_10_ (p-value)

Functional enrichment analysis of the PSGs using Gene Ontology Biological Process terms yielded patterns partially consistent with the gene-level findings (Fig. 2, Supplementary file 3). *I. geoffrensis* and *L. vexillifer*, or *I. geoffrensis* and *S. fluviatilis*, did not share enriched terms, despite phylogenetic relatedness, whereas *L. vexillifer* and *N. asiaeorientalis*, as well as *N. asiaeorientalis* with *P. minor* and *P. gangetica*, shared some biological processes such as hemopoiesis, skeletal system development, embryonic morphogenesis, head development, and skeletal system development. *N. asiaeorientalis* showed extensive functional overlap, sharing three GO terms with *L. vexillifer*, and five each with *P. minor* and *P. gangetica*. Interestingly, *P. minor* and *P. gangetica*, which are sister species, shared only one enriched function, highlighting that adaptive responses at the functional level are not uniform, even among closely related species.

An additional enrichment analysis based on PSGs identified when all freshwater lineages were jointly specified as foreground branches (Supplementary Fig. S4) recovered several biological processes that were also detected in the pairwise and multi-species comparisons, including neuron projection development, hemopoiesis, import into cell, cell junction organization, pattern specification process, regulation of cellular response to stress, skeletal system development, modulation of chemical synaptic transmission, enzyme-linked receptor protein signaling pathway, and embryo development ending in birth or egg hatching. This overlap indicates that a subset of the functional signals observed across independent lineage comparisons is also detected when freshwater taxa are analyzed collectively.

Together, these results show that while widespread convergence across all riverine cetaceans is not apparent, subsets of species exhibit recurring gene-level and functional overlap. These patterns are most evident in species from Asia and are primarily observed in pairwise and small-group comparisons. Adaptive convergence in this system appears to be partial and context-dependent, with statistical analyses indicating that convergence is best explained by the interaction between shared ancestry and ecological similarity, rather than by either factor alone.

### Patterns of Expanded and Contracted Gene Families

To evaluate whether freshwater adaptation is also reflected in changes in gene family composition, we next investigated lineage-specific expansions and contractions across riverine cetaceans. Gene family evolution was analyzed using CAFE across a total of 17,752 gene families. Using a significance threshold of p < 0.01, we identified 845 gene families showing significant expansion or contraction. Based on the number of tests performed, we estimated that approximately 178 gene families would be expected to be significant by chance alone. Thus, the observed number of significant families exceeds random expectations, suggesting that most detected changes likely reflect true evolutionary signals.

**Fig. 3:**
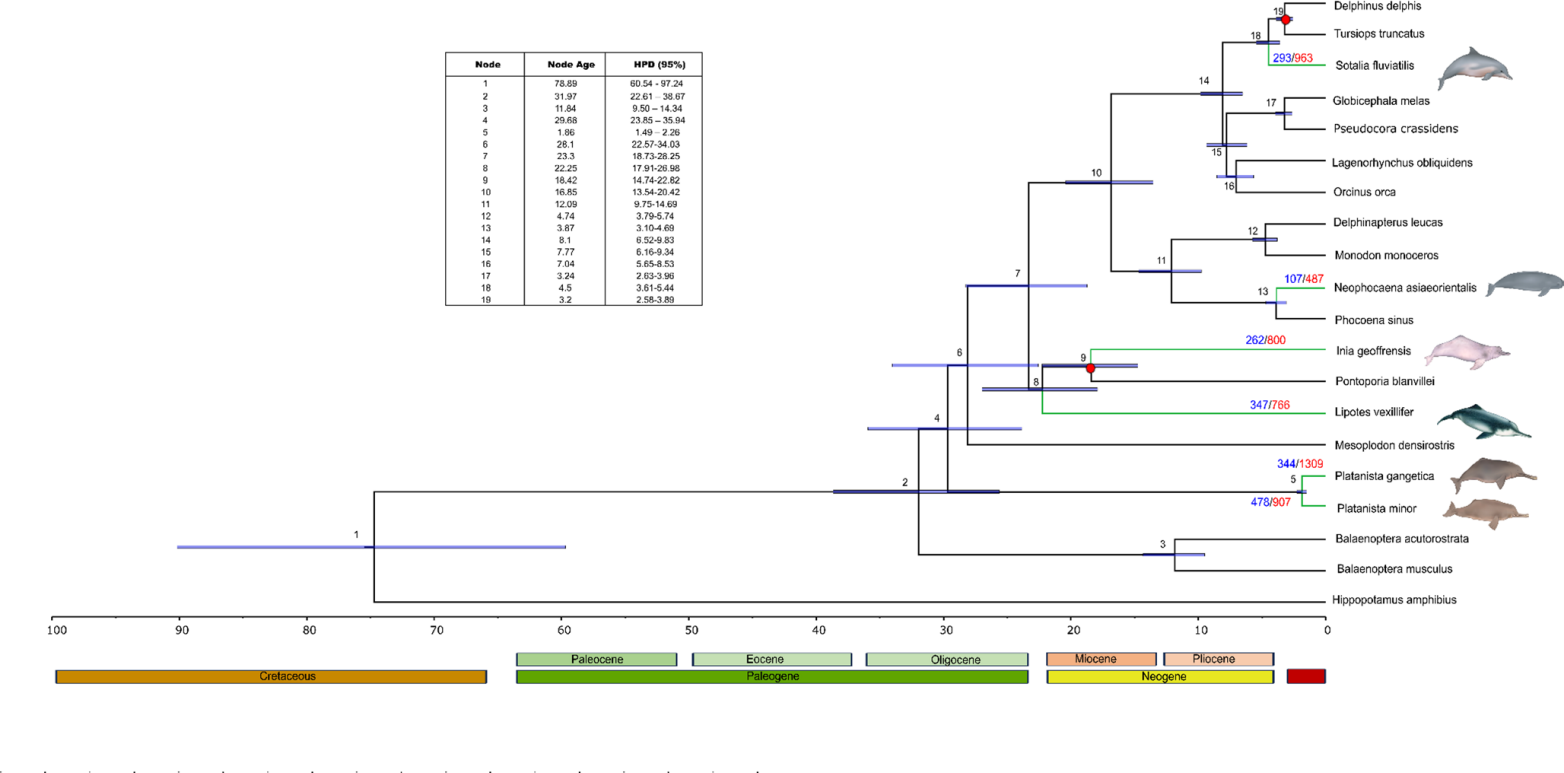
Gene family expansions and contractions across riverine lineages mapped onto a time-calibrated phylogenetic tree constructed using BEAST. Numbers at each branch indicate gene family contractions (blue) and expansions (red) for the respective lineages. Red dots on the nodes correspond to calibration points. Iniidae and Pontoporidae were calibrated with the fossil Brachydelphis and Delphininae with the fossil Eodelphinus kabatensis (Supplementary file 1. Table S3). The table on the left shows the 95% HPD values (Color figure online)

**Fig. 4:**
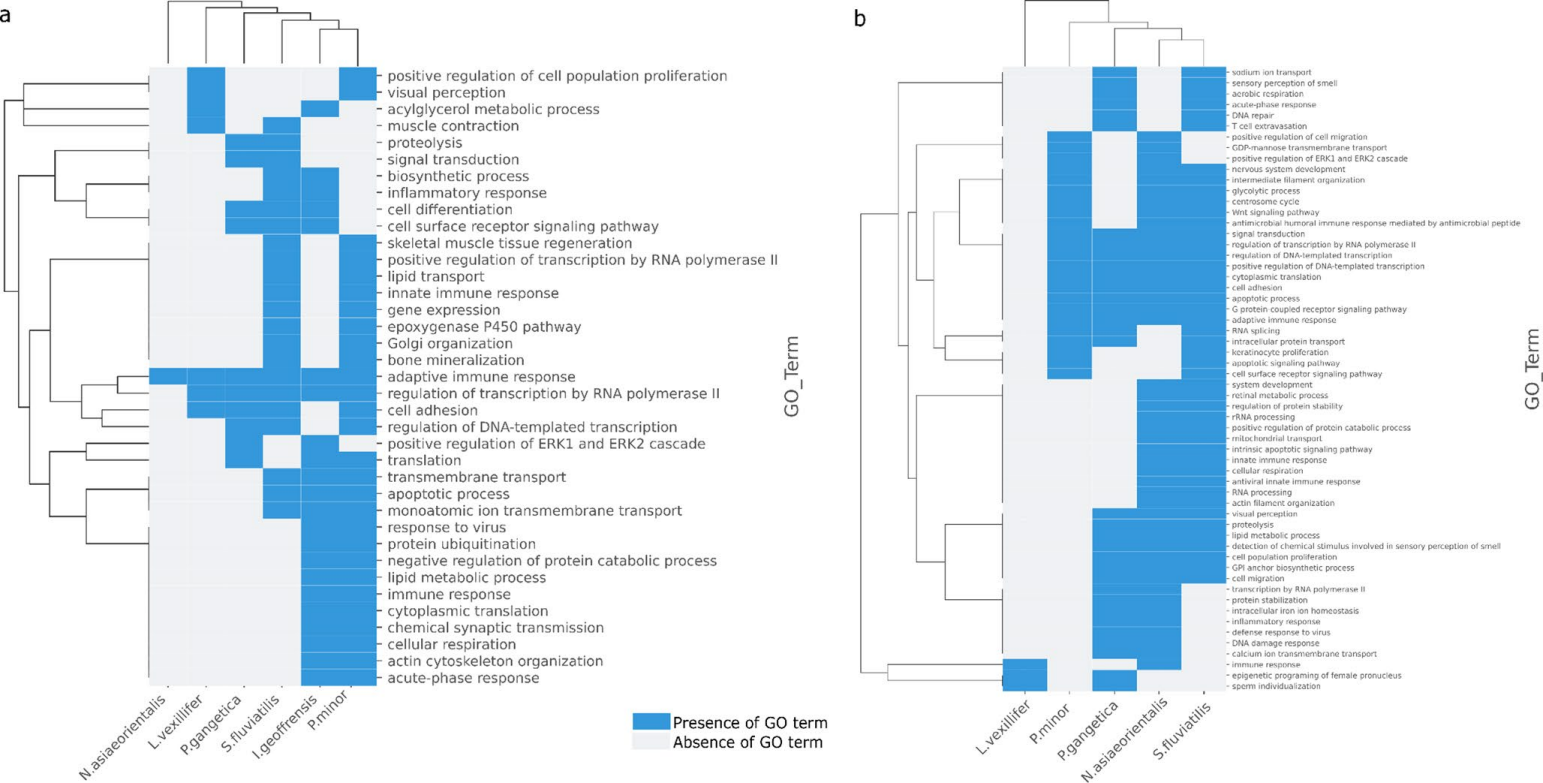
Heatmap and dendrogram illustrating Gene Ontology (GO) biological process terms associated with gene family expansions and contractions in river dolphin lineages. **a** GO terms associated with gene family expansions shared among river dolphin lineages. **b** GO terms associated with gene family contractions shared among river dolphin lineages. The dendrograms above each heatmap represent the hierarchical clustering of species based on the similarity of their GO term profiles. The dendrograms to the left of each heatmap cluster GO terms based on their distribution across the different species. Blue cells indicate the presence of a GO term in a given species, while white cells indicate its absence (Color figure online)

To investigate the biological relevance of gene family size variation, we performed gene ontology (GO) analyses on both expanded and contracted gene families across six species: four from Asia (*N. asiaeorientalis*, L. vexillifer, *P. gangetica, P. minor)* and two from the Americas (*I. geoffrensis* and *S. fluviatilis*) (Fig. 3). GO terms were visualized using heatmaps with hierarchical clustering (Fig. 4, Supplementary file 4), enabling a comparative assessment of functional patterns linked to gene family expansions and contractions.

The analysis of GO terms associated with gene family expansions and contractions revealed a subset of biological functions recurrent across most freshwater dolphin species, regardless of lineage or geography. Notably, adaptive immune response, transcriptional regulation by RNA polymerase II, and cell adhesion were present in both expanded and contracted gene families across multiple species (Fig. 4). This suggests that these processes are under dynamic evolutionary pressures, potentially involving both gene gain and loss events.

For gene family expansions, hierarchical clustering of enriched GO terms revealed no apparent phylogenetic or geographic grouping (Fig. 4). Asian and American species clustered together, suggesting that expansions likely result from lineage-specific selective pressures. Among the enriched functions, processes such as innate immune response, lipid transport, and signal transduction were also frequently observed, whereas others, like response to virus and Golgi organization, were more species-specific. For gene family contractions, a greater functional diversity and more pronounced lineage specificity were observed. Importantly, *Inia geoffrensis* did not cluster with any other species in the contraction dataset, as it lacked shared GO terms in this category. Among the remaining species, contractions were enriched in processes related to RNA processing, cytoplasmic translation, and immune regulation. As with expansions, some immune-related terms, such as adaptive immune response and T cell extravasation, appeared across several species, suggesting common patterns of immune modulation.

### Convergent Evolutionary Rates

**Fig. 5:**
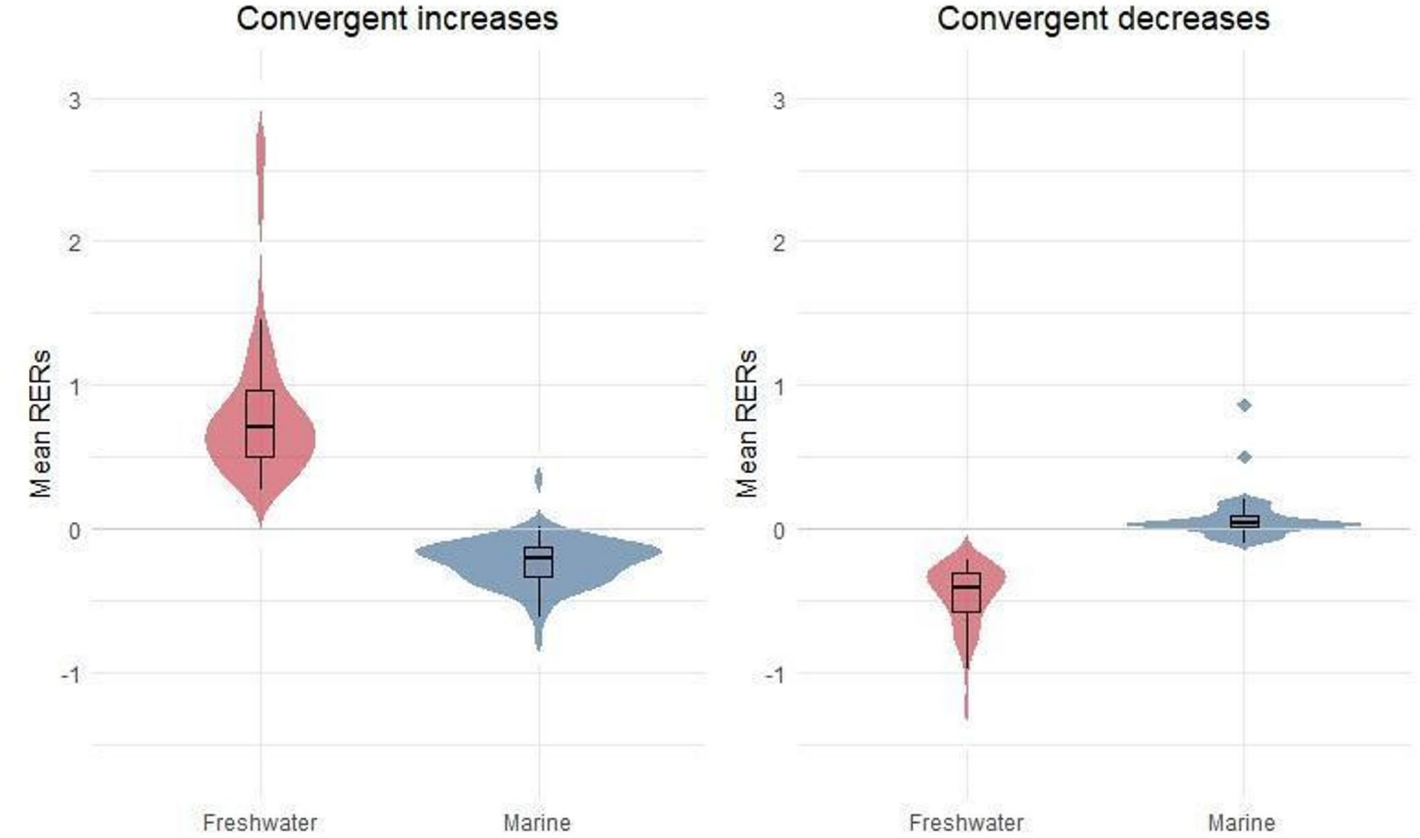
Violin plots depicting the distribution of mean convergent relative evolutionary rates (RERs) in freshwater (pink) versus marine (blue) species. Convergent rate increases and decreases are shown in the left and right panels, respectively. Data are filtered to include only statistically significant convergent shifts (p ≤ 0.01) (Color figure online)

To detect genes exhibiting consistent shifts in evolutionary rates across independent freshwater transitions, we performed an analysis of convergent evolutionary rates using RERconverge. RERconverge analyses identified 95 genes with convergent evolutionary rate shifts in freshwater cetaceans relative to marine species (Fig. 5, Supplementary Fig. S3). Among these, 64 genes had convergently increased relative evolutionary rates, and the other 31 had convergently decreased rates compared to their marine counterparts (Supplementary Table S7).

Enriched GO terms among genes with convergent rates (increased and decreased) highlighted biological processes associated with cellular regulation, including cell number homeostasis, nitrogen compound response, transcription factor regulation, RNA polymerase II elongation, and growth factor signaling (Supplementary Fig. S1), suggesting repeated molecular responses to freshwater environmental pressures.

An integrated GO analysis combining convergent genes and shared PSGs (35 genes in total) recovered several pathways repeatedly enriched across independent analyses (Supplementary Table S8). These results point to a core subset of molecular functions consistently associated with freshwater specialization in odontocetes.

## Discussion

This study investigated the genomic signatures of freshwater adaptation across six independent cetacean lineages through integrated analyses of positive selection, gene family dynamics, and evolutionary rate convergence. Our results reveal that freshwater colonization in cetaceans involves convergent evolution of fundamental biological systems, including immune response, osmoregulation, skeletal development, and sensory processing, though these adaptations do not follow a universal genomic program across all lineages.

We emphasize that gene-level convergence inferred from branch-site analyses should be interpreted cautiously. As noted in the Results, these signals represent putative convergence, reflecting recurrent lineage-specific positive selection across freshwater taxa rather than formal statistical evidence of independent convergent selection events. This distinction is important for correctly interpreting the evolutionary patterns discussed below.

Despite analyzing six freshwater species spanning distinct phylogenetic lineages and geographic regions, we found no genes under positive selection shared by all species and no evidence of a single, consistent clade-level enrichment of positively selected genes. Instead, convergence emerged at functional rather than gene levels, with Asian species (*L. vexillifer, N. asiaeorientalis, P. gangetica, P. minor*) showing higher gene-level overlap than American taxa (*I. geoffrensis, S. fluviatilis*). This regional pattern is supported by statistical comparisons (Welch’s t-test, p = 0.044), whereas phylogenetic relatedness and shared habitat alone were not significant predictors of PSG overlap. This pattern suggests that while similar ecological pressures drive adaptation to freshwater environments, multiple molecular pathways can achieve comparable functional outcomes, a hallmark of convergent evolution in complex traits.

### Core Biological Systems Under Selection

Gene Ontology enrichment analyses revealed four major biological systems consistently targeted by selection across freshwater lineages, reflecting the physiological challenges imposed by riverine habitats: (i) immune system adaptation, (ii) osmoregulation and cellular homeostasis, (iii) craniofacial and skeletal development and (iv) neuromuscular coordination. Regarding the immune system adaptation, the enrichment of “hematopoiesis” and “adaptive immune response” terms across multiple species suggests strong selective pressure from freshwater pathogen communities. Freshwater cetaceans are known to experience distinct and often higher pathogen exposure, with documented cases of infectious lesions, pneumonia, parasitic infections, and bacterial disease in river dolphins such as *Inia geoffrensis* and *Platanista gangetica* (Pier et al. 1978; Sutaria et al. 2019; Campbell et al. 2022). Positively selected genes such as HDAC5 (hematopoietic stem cell regulation) and RHAG (erythrocyte integrity) in *L. vexillifer, P. gangetica,* and *N. asiaeorientali*s indicate that blood cell production and immune modulation have been recurrent evolutionary targets in these taxa. This pattern is consistent with the idea that distinct pathogen regimes in freshwater systems can shape mammalian immune genes, as suggested for TLR-mediated responses in riverine manatees (Oliveira et al. 2021), but direct comparative physiological or immunological data specifically contrasting freshwater and marine cetaceans are not yet available.

Selection on genes associated with osmoregulation and cellular homeostasis reflects adaptations to hypo-osmotic stress characteristic of freshwater systems, and previous comparative analyses have already identified signatures of selection in osmoregulatory pathways (e.g., AVP, AQP2, SLC14A2) in riverine cetaceans relative to marine taxa (Ramos et al. 2023). In odontocetes, such departures from marine salinity have well‑documented physiological consequences, with prolonged freshwater exposure in bottlenose dolphins leading to hyponatremia, hypochloremia, altered serum osmolality, and skin lesions (Ewing et al. 2017). Similarly, SNPs identified in genes related to osmotic regulation were associated with adaptation to freshwater environments in Pacific herring (Nedoluzhko et al. 2022) and Atlantic salmon (Harder and Christie 2022) populations, indicating that changes in osmoregulation are essential to the marine-freshwater transition in distinct vertebrate lineages. In this context, the enrichment of GO terms "import to cell" and "cell junction organization" in multiple species highlights the importance of maintaining ionic balance in environments with fluctuating salinity. SLC6A12, under positive selection in *P. gangetica* and *P. minor*, transports osmolytes like betaine that are critical for osmotic stress mitigation (Xu et al. 2013). Similarly, TRPV4, selected in *L. vexillifer*, *N. asiaeorientalis*, and *P. minor*, encodes an ion channel responsive to cell volume changes, osmotic pressure, and mechanical stress, contributing to tight junction maintenance in kidney and neural tissues essential for epithelial integrity and ionic balance (Tokuda and Yu 2019; Xin and Yanlong 2024). These molecular signatures are therefore consistent with the hypothesis that osmoregulatory genes have been recurrent targets of selection during freshwater colonization in cetaceans, while their specific functional roles in riverine species remain to be experimentally tested.

Craniofacial and skeletal development emerged as another key adaptive system, with enrichment of terms such as “skeletal system development,” “head development,” and “embryonic morphogenesis” in *N. asiaeorientalis* and *P. gangetica*. Morphological studies on river dolphins show that these species possess elongated rostra, mediolaterally compressed skulls, and modified cervical and thoracic regions that enhance maneuverability and prey capture in shallow, structurally complex, and often turbid river channels, supporting a link between cranial remodeling and the hydrodynamic and sensory constraints of riverine habitats (Hamilton et al. 2001). Convergent evolution in cranial morphology across phylogenetically distant river dolphin lineages has been well documented (Page and Cooper 2017), and fossil evidence from *Pebanista yacuruna* indicates that elongated skulls and supraorbital crests arose early in riverine odontocetes, consistent with repeated selection on craniofacial traits in these environments (Benites-Palomino et al. 2024). Genes under positive selection in *N. asiaeorientalis* and *P. gangetica*, including FOXC1 and DLX6 (craniofacial development regulators) and HOXB3 (pharyngeal arch patterning), therefore represent plausible candidates contributing to these skeletal changes, although their specific roles in cetacean skull evolution remain hypothetical and will require functional validation (Robledo et al. 2002; Zhang et al. 2021). The observation that skeletal development genes are recurrently targeted by selection in other ecologically specialized mammals, including freshwater mammals like *Trichechus senegalensis* (Huang et al. 2024) and terrestrial species like *Equus ferus caballus* (Kader et al. 2016; Salek et al. 2019) and the rodent *Tupaia belangeri chinensis* (Yu et al. 2014), further suggests that modulation of bone growth, patterning, and remodeling is a common route by which vertebrates achieve morphological adaptation to novel environments.

Finally, selection on synaptic transmission pathways may reflect adaptations to the physical demands of riverine navigation, where fine control of body posture and propulsion is required in shallow, heterogeneous, and fast‑flowing channels. The enrichment of “synaptic transmission modulation” in *N. asiaeorientalis* and *P. minor*, together with the detection of positive selection in MUSK and LRP4—key regulators of neuromuscular junction formation and acetylcholine receptor clustering (Herbst 2020; Ohkawara et al. 2020)—is consistent with the hypothesis that neuromuscular signaling has been tuned to support rapid, precise muscle coordination in these species. Although comparable kinematic and electrophysiological data are not yet available for river dolphins, biomechanical and anatomical work in other cetaceans and large aquatic vertebrates shows that maneuverability and fine motor control rely critically on neuromuscular specialization and coordinated activation of axial and appendicular musculature (Barik et al. 2014; Burden et al. 2015), which provides a functional context for interpreting the observed genomic signals while highlighting the need for future integrative studies.

### Widely Shared Genes Under Selection

Beyond functional convergence, we identified 16 genes showing positive selection in at least four of six species, with NSMAF and CTRL uniquely selected in five species (Table 1). The convergent selection of these genes across phylogenetically distant and geographically separated lineages is particularly noteworthy, suggesting they represent constrained solutions to fundamental freshwater challenges. NSMAF, involved in TNF-α signaling and ceramide metabolism, may play a critical role in managing inflammatory responses associated with increased muscle stress in riverine environments. Swimming in rivers imposes hydrodynamic conditions distinct from the ocean, including irregular currents, turbulence, and reduced buoyancy, requiring frequent directional changes and rapid responses to obstacles. Selection on NSMAF may have enhanced the capacity to modulate inflammation and maintain muscle integrity under these demanding locomotor conditions (Jung et al. 2022). CTRL, encoding the digestive enzyme chymotrypsin-like serine protease involved in trypsinogen degradation (Reseland et al. 1997; Eiseler et al. 2023), could potentially reflect specific digestive adaptations that might be necessary for riverine life. Considering the differences in prey availability, size, and nutritional composition between marine and freshwater ecosystems, the selection of CTRL might indicate functional modifications in protein digestion that could be necessary for efficiently exploiting riverine food resources, which may include different fish species, crustaceans, or invertebrates with varying protein structures compared to typical marine prey.

**Table 1:** Genes under positive selection shared among four and five species

Species count	Gene	Functional role
4	ARL9	GTPase involved in intracellular vesicle trafficking and signal transduction (Donaldson and Jackson 2011)
4	HDAC5	Histone deacetylase that regulates gene expression and is involved in muscle and neuronal development (Agis-Balboa et al. 2013)
4	MNX1	Transcription factor essential for motor neuron development and pancreatic cell differentiation (Tian et al. 2018)
4	EML2	Microtubule-associated protein that regulates cytoskeleton organization and cell division (Hotta et al. 2022)
4	HIP1	Involved in clathrin-mediated endocytosis and membrane trafficking processes (Hyun and Ross 2004)
4	INPP5B	Phosphatase that hydrolyzes phosphoinositides, regulating cellular signaling pathways (Droubi et al. 2022)
4	GRIN3B	NMDA receptor subunit that modulates synaptic transmission and neuronal excitability (Hornig et al. 2017)
4	PPP1R12B	Regulatory subunit of myosin phosphatase involved in smooth muscle contraction (Montén et al. 2015)
4	SH2D6	Adaptor protein involved in tyrosine kinase signaling pathways (Wu et al. 2023)
4	PARP10	Mono-ADP-ribosyltransferase involved in transcriptional regulation and DNA repair (Shahrour et al. 2016)
4	BMPR1B	BMP receptor that regulates bone and cartilage development through TGF-β signaling (Yoon et al. 2005)
4	ETV2	Master transcription factor for vascular and blood cell development (Oh et al. 2015)
4	ANKRD23	Ankyrin repeat protein involved in transcriptional regulation and metabolic processes (Shimoda et al. 2015)
4	CNMD	Regulator of chondrocyte growth with antiangiogenic activity in cardiovascular development (Reyes et al. 2024)
5	NSMAF	Adaptor protein linking TNF receptor signaling to sphingomyelin metabolism (Jung et al. 2022)
5	CTRL	Pancreatic digestive enzyme with chymotrypsin-like proteolytic activity (Eiseler et al. 2023)

The genes shared under positive selection among the four species (Table 1) span diverse biological processes yet converge on interconnected functions related to structural organization, physiological regulation, and immune defense. BMPR1B, CNMD, EML2, HIP1, PPP1R12B, and ANKRD23 are functionally linked to structural organization and tissue development, coordinating cytoskeletal dynamics, membrane trafficking, and musculoskeletal formation (Yoon et al. 2005; Reyes et al. 2024; Hotta et al. 2022; Hyun and Ross 2004; Montén et al. 2015; Shimoda et al. 2015). MNX1, GRIN3B, ARL9, and HDAC5 are jointly associated with neuronal differentiation and signaling, contributing to synaptic modulation, intracellular transport, and transcriptional control (Agis-Balboa et al. 2013; Hornig et al. 2017; Tian et al. 2018; Donaldson and Jackson 2011). INPP5B and ETV2 play central roles in maintaining physiological homeostasis through the regulation of phosphoinositide signaling and vascular or hematopoietic differentiation (Oh et al. 2015; Droubi et al. 2022). Finally, PARP10 and SH2D6 are implicated in immune signaling and cellular defense, integrating DNA repair mechanisms with tyrosine kinase–mediated pathways (Shahrour et al. 2016; Wu et al. 2023).

This functional convergence in distantly related species suggests the possibility that adapting to freshwater environments may have required the remodeling of fundamental metabolic and physiological pathways, though the exact mechanisms remain to be fully understood. Also, the convergent selection of these genes in phylogenetically distant and geographically separated species suggests potential shared evolutionary pathways toward freshwater life, though the specific adaptive mechanisms remain to be fully elucidated.

### Gene Family Dynamics

CAFE analyses revealed dynamic patterns of gene family expansions and contractions, with three functional categories appearing in both expanded and contracted gene families across most species: “adaptive immune response”, “transcriptional regulation by RNA polymerase II”, and “cell adhesion”. The presence of both expansions and contractions within the same functional categories indicates that riverine adaptation involves not only enhancing certain capabilities through gene duplication but also streamlining others through gene loss, optimizing the genomic repertoire for freshwater challenges (Lynch and Conery 2000; Hahn 2009).

The recurrence of “adaptive immune response” across both positive selection analyses and gene family dynamics underscores the importance of immune system remodeling for successful freshwater colonization. Freshwater ecosystems likely present pathogen loads and microbial communities distinct from marine systems, with potentially different transmission dynamics and host–pathogen interactions (Romano et al. 1992). Genetic variability in immune-related genes may facilitate adaptation to novel pathogens specific to riverine ecosystems (Vazquez et al. 2024), and positive selection in immune genes has been observed across various mammals, including cetaceans (Sá et al. 2019; Zhang et al. 2019; Dias and Nery 2020; Schaschl and Wallner 2020; Day et al. 2024). Similar patterns of immune gene family expansion and contraction have been documented in primates, rodents, bats, and freshwater fish (Teekas et al. 2022; Garg et al. 2025; Liu et al. 2021), suggesting that immune system evolution is a general feature of adaptation to novel environments. For instance, genomic analyses of the white-blotched river stingray (*Potamotrygon leopoldi*), a species whose ancestor experienced a similar marine-to-freshwater transition, revealed a significant expansion of immune-related gene families (Zhou et al. 2023). Likewise, the roughskin sculpin (*Trachidermus fasciatus*), a diadromous fish that migrates between marine and freshwater habitats, showed evidence of gene family expansion and positive selection on immune response–related genes (Liu et al. 2025).

The enrichment of “transcriptional regulation by RNA polymerase II” in both expansions and contractions may reflect the need for flexible gene expression responses to variable environmental conditions characteristic of freshwater systems, including seasonal changes in water chemistry, temperature fluctuations, and pollution levels. Alterations in transcription speed can influence RNA processing and potentially generate adaptive functional variants necessary for surviving in dynamic riverine environments (Muniz et al. 2021). Similarly, the recurrent enrichment of cell adhesion–related functions across freshwater lineages suggests that epithelial and tissue integrity may represent an important target of selection in riverine habitats. In aquatic vertebrates, transitions from marine to freshwater environments impose strong hypo-osmotic stress, requiring extensive remodeling of epithelial barriers involved in osmoregulation and ion homeostasis. Comparative genomic analyses in fishes have shown that genes associated with cell adhesion molecules and tight junction pathways are consistently enriched among loci underlying freshwater versus marine adaptation, highlighting the importance of epithelial adhesion and barrier function in low-salinity environments (Qian et al. 2025). Importantly, genomic evidence from riverine cetaceans supports a similar pattern. Zhou et al. (2013) reported expansion of cell adhesion–related gene families in the genome of the freshwater baiji (*Lipotes vexillifer*), and Yuan et al. (2018) independently identified expansion of adhesion and junction-associated gene families in the Yangtze finless porpoise (*Neophocaena asiaeorientalis*). Together with our results, these independent findings suggest that repeated remodeling of cell adhesion pathways represents a recurrent genomic response to freshwater colonization in cetaceans, which may reflect selective pressures acting on epithelial tissues exposed to sustained hypo-osmotic conditions.

Interestingly, our results show that the GO term “visual perception” is associated with gene family contraction in *P. gangetica, N. asiaeorientalis,* and *S. fluviatilis*. Similarly, Actinopterygii species inhabiting darker environments have been shown to possess reduced repertoires of opsin genes (Policarpo et al. 2025). In contrast, *L. vexillifer* and *P. minor* exhibited expansions of gene families related to visual perception. These divergent patterns may be related to environmental heterogeneity, such as differences in habitat depth. For example, freshwater and shallow-water ray-finned fishes possess a higher number of SWS1 (short-wavelength-sensitive opsin) and LWS (long-wavelength-sensitive opsin) genes per species than their marine counterparts (Lin et al. 2017), and LWS genes are commonly lost in deep-sea teleost lineages (Musilova et al. 2019).

Hierarchical clustering based on enriched GO terms from gene family dynamics did not reflect clear phylogenetic or geographic structure, with Asian and American species clustering together in both expansion and contraction analyses. This pattern supports the interpretation that gene family evolution reflects independent selective responses to similar freshwater pressures rather than shared ancestry or parallel evolution. Notably, *I. geoffrensis* did not cluster with any other species in the contraction dataset, as it lacked shared GO terms in this category, highlighting substantial variation in evolutionary trajectories even among closely related freshwater specialists.

### Convergent Evolutionary Rates

RERconverge analysis identified 95 genes with convergent evolutionary rate shifts associated with freshwater adaptation, enriched in biological processes including cell number homeostasis, nitrogen compound response, transcription factor regulation, RNA polymerase II elongation, and growth factor signaling. While these results should be interpreted cautiously, given that no genes survived multiple testing correction, the functional enrichment patterns suggest repeated molecular responses to freshwater environmental pressures across independent lineages.

The enrichment of the GO term “cellular response to nitrogen compounds” is particularly intriguing, as freshwater environments present different concentrations of ammonia, urea, and other nitrogenous products compared to marine systems (Rosenwasser et al. 2014). Genes identified under evolutionary convergence associated with this GO term include GABRB1 and GCLM, where GCLM plays a fundamental role in glutamate metabolism and glutathione synthesis, key enzymes for assimilating inorganic nitrogen into organic compounds (Bernard et al. 2009). Glutamate/glutamine catabolism facilitates acute osmotic adjustment in branchial cells through the supply of energy and nitrogenous waste (Liew et al. 2020), which aligns with documented physiological challenges faced by freshwater aquatic mammals and other riverine vertebrates, including increased detoxification demand, and osmoregulatory regulation associated with nitrogenous waste exposure in low-salinity environments (Campbell et al. 2022; Wang et al. 2022; He et al. 2023).

Integration of genes showing convergent rate shifts with positively selected genes shared across multiple lineages yielded 34 genes total, with enrichment analyses recovering several pathways critical for freshwater adaptation. Notable enriched terms included “visual perception” and “sensory perception of sound” associated with EML2 (Lepley et al. 1999). Although primarily described in humans, this gene may be important in riverine adaptation due to significant differences between marine and freshwater environments, the latter characterized by a highly dynamic, turbid, and acoustically complex environment, reducing visibility and complicating prey detection (Choudhury et al. 2019; Aggarwal et al. 2020; Xie et al. 2025). Genetic convergence linked to “visual perception” has been reported in the river dolphin *Lipotes vexillifer* (Zhou et al. 2013), some primates, subterranean rodents (Indrischek et al. 2022), and bats (Hudson et al. 2014), suggesting convergent reduction or modification of visual function in low-light or challenging sensory environments. Similarly, genes related to visual perception are under selection in fish that colonized freshwater environments. Positive selection on South American freshwater croaker rhodopsin (and its consequent red-shift when compared to its marine ancestor) has been associated with the marine-to-freshwater transition (Van Nynatten et al. 2021). Sites under positive selection in the same gene have also been identified in freshwater anchovy lineages with marine ancestors (Van Nynatten et al. 2015). Furthermore, amino acid polymorphisms that may cause shifts in light absorption were associated with the postglacial colonization of lakes with varying light environments by threespine sticklebacks (Marques et al. 2017).

Similarly, convergence in “sensory perception of sound” genes has been documented in pangolins and bats (Dong et al. 2016; Li et al. 2024), supporting our findings and highlighting shared adaptations to demanding sensory landscapes.

## Conclusion and Future Directions

Our comparative genomic analyses demonstrate that freshwater adaptation in cetaceans involves putative convergent evolution of multiple fundamental biological systems rather than a single, shared set of genomic changes. The absence of clear phylogenetic or geographic patterns in gene-level convergence, coupled with functional convergence and lineage-specific signals, indicates that similar ecological pressures can be met through diverse molecular solutions, a pattern reflecting the inherent complexity of adaptive evolution in mammals. The identification of shared genes under selection highlights critical pathways that may represent functionally constrained solutions to freshwater challenges. Gene family dynamics reveal active genomic remodeling through both expansions and contractions, emphasizing that adaptation involves optimization rather than simple addition of function. Taken together, our results suggest that freshwater adaptation is shaped by an interplay of shared ancestry, local environmental pressures, and lineage-specific evolutionary histories. Future research incorporating functional analyses and gene expression data under different environmental conditions will be essential to validate these adaptive hypotheses and deepen our understanding of convergent evolution in aquatic mammals.

## Supplementary Information

Below is the link to the electronic supplementary material.

Supplementary file1 (DOCX 321 kb)

Supplementary file2 (XLSX 2562 kb)

Supplementary file3 (XLSX 34 kb)

Supplementary file4 (XLSX 41 kb)

Supplementary file5 (XLSX 18 kb)

## Data Availability

All genome data used in this study were obtained from the NCBI databases (Supplementary Table 1).
