# Supplementary material for: Convergent Functional Genomic Evolution Underlying Repeated Freshwater Colonization in Cetaceans
Source: J Mol Evol. 2026 Jun 22;94(4):596–614. doi: 10.1007/s00239-026-10325-4 (PMC13433582; doi:10.1007/s00239-026-10325-4)
Supplement: Supplementary file 1 [file 239_2026_10325_MOESM1_ESM.docx]

**Supplementary material**

**Genomic Signatures of Freshwater Adaptation in Riverine Cetaceans: A Comparative Evolutionary Analysis**

**Journal of Molecular Evolution**

D Pinilla-Beltrán, Leonardo Duarte-Santos, Mariana F Nery

Laboratory of Evolutionary Genomics. Department of Genetics, Evolution, Immunology and Microbiology, University of Campinas, SP 13083-862, Brazil.

Corresponding author: Mariana F. Nery, PhD, Laboratório de Genômica Evolutiva. Departamento de Genética, Evolução, Microbiologia e Imunologia, Universidade Estadual de Campinas, Cidade Universitária, 13083970 - Campinas, SP -Brazil.

**E-mail:** [marinery@unicamp.br](mailto:marinery@unicamp.br)

**Supplementary Table 1** Species included in the study. The first column refers to the suborder to which each species belongs, the second to the scientific name of the species, the third to the common name of the species, and the fourth to the Genbank code referring to the downloaded genome.

| **Suborder** | **Species** | **Common name** | **GenBank code** |
| --- | --- | --- | --- |
| Odontoceti | *Delphinapterus leucas* | Beluga | GCA_002288925.3 |
| Odontoceti | *Delphinus delphis* | Short-beaked saddleback dolphin | GCA_949987515.1 |
| Odontoceti | *Globicephala melas* | Long-finned pilot whale | GCA_963455315.1 |
| Odontoceti | *Inia geoffrensis* | Boto | GCA_036417435.1 |
| Odontoceti | *Lagenorhynchus albirostris* | White-beaked dolphin | GCA_949774975.1 |
| Odontoceti | *Lipotes vexillifer* | Baiji | GCA_000442215.2 |
| Odontoceti | *Mesoplodon densirostris* | Beaked whale | GCA_025265405.1 |
| Odontoceti | *Monodon monoceros* | Narwhal | GCA_005190385.2 |
| Odontoceti | *N. asiaeorientalis asiaeorientalis* | Yangtze finless porpoise | GCA_003031525.2 |
| Odontoceti | *Orcinus orca* | killer whale | GCA_937001465.1 |
| Odontoceti | *Phocoena sinus* | Cochito | GCA_008692025.1 |
| Odontoceti | *Platanista minor* | Indus river dolphin | GCA_004363435.1 |
| Odontoceti | *Platanista gangetica* | Susu | GCA_017311385.1 |
| Odontoceti | *Pontoporia blainvillei* | Franciscana | GCA_011754075.1 |
| Odontoceti | *Sotalia fluviatilis* | Tucuxi | SUB15967528 |
| Odontoceti | *Tursiops truncatus* | Bottlenosed dolphin | GCA_011762595.1 |
| Misticeti | *Balaenoptera acutorostrata* | Common minke whale | GCA_949987535.1 |
| Misticeti | *Balaenoptera musculus* | Blue whale | GCA_009873245.3 |
| Whippomorpha | *Hippopotamus amphibius* | Hippopotamus | GCA_030028045.1 |

**Supplementary Table 2** Busco evaluation *of Inia geoffrensis, Pontoporia blainvillei,* *Platanista gangetica, Platanista minor, Sotalia fluviatilis* annotation

| **Inia geoffrensis** | | | | |
| --- | --- | --- | --- | --- |
| C:94.2%[S:93.4%,D:0.8%],F:0.5%,M:5.3%,n:9226 | | | | |
| Complete BUSCOs (C) | | | | 8686 |
| Complete and single-copy BUSCOs (S) | | | | 8615 |
| Complete and duplicated BUSCOs (D) | | | | 71 |
| Fragmented BUSCOs (F) | | | | 45 |
| Missing BUSCOs (M) | | | | 495 |
| Total BUSCO groups searched | | | | 9226 |
|  |  |  |  |  |
| **Platanista gangetica** | | | | |
| C:86.9%[S:86.5%,D:0.4%],F:2.4%,M:10.7%,n:9226 | | | | |
| Complete BUSCOs (C) | | | | 8024 |
| Complete and single-copy BUSCOs (S) | | | | 7983 |
| Complete and duplicated BUSCOs (D) | | | | 41 |
| Fragmented BUSCOs (F) | | | | 226 |
| Missing BUSCOs (M) | | | | 976 |
| Total BUSCO groups searched | | | | 9226 |
|  |  |  |  |  |
| **Pontoporia blanvillei** | | | | |
| C:92.1%[S:91.4%,D:0.7%],F:1.4%,M:6.5%,n:9226 | | | | |
| Complete BUSCOs (C) | | | | 8506 |
| Complete and single-copy BUSCOs (S) | | | | 8437 |
| Complete and duplicated BUSCOs (D) | | | | 69 |
| Fragmented BUSCOs (F) | | | | 133 |
| Missing BUSCOs (M) | | | | 587 |
| Total BUSCO groups searched | | | | 9226 |

| **Platanista minor** | |
| --- | --- |
| C:93.5%[S:92.0%,D:1.5%],F:1.2%,M:5.3%,n:9226 | |
| Complete BUSCOs (C) | 8624 |
| Complete and single-copy BUSCOs (S) | 8486 |
| Complete and duplicated BUSCOs (D) | 138 |
| Fragmented BUSCOs (F) | 110 |
| Missing BUSCOs (M) | 429 |
| Total BUSCO groups searched | 9226 |

| **Sotalia fluviatilis** | |
| --- | --- |
| C:96.3%[S:95.1%,D:1.2%],F:0.9%,M:2.8%,n:9226 | |
| Complete BUSCOs (C) | 8885 |
| Complete and single-copy BUSCOs (S) | 8778 |
| Complete and duplicated BUSCOs (D) | 107 |
| Fragmented BUSCOs (F) | 81 |
| Missing BUSCOs (M) | 260 |
| Total BUSCO groups searched | 9226 |

**Supplementary Table 3** Fossil calibrations used in the divergence time estimation. Age intervals are represented in millions of years ago (Mya). Node numbers correspond to those in Figure 1.

| **Node number** | **Description** | **Age interval  (My)** | **Fossil calibration** | **Citation** |
| --- | --- | --- | --- | --- |
| A | Inoidea | 11.2- 16.6 | *Brachydelphis* | [Werdelin (1989)](https://pascal-francis.inist.fr/vibad/index.php?action=getRecordDetail&idt=PASCALGEODEBRGM8320343197) |
| B | Delphininae | 3.98 - 8.5 | *Etruridelphis giulii* | Bianucci (2013); Murakami et al. (2014) |

**Supplementary Table 7** Gene Ontology analysis of 34 genes associated with riverine adaptation, including positively selected genes and genes with evolutionary rate changes identified through RERconverge analysis.

| **SP** | **GENE_NAME** | **UniProtKB_ID** | **GO_TERM** | **GO Term - Biological funtion** |
| --- | --- | --- | --- | --- |
| Homo sapiens | FOXJ2 | Q9P0K8 | GO:0030154 | cell differentiation |
| Homo sapiens | CASTOR2 | A6NHX0 | GO:1903577 | cellular response to L-arginine |
| Homo sapiens | MACROD1 | Q9BQ69 | GO:0006974 | DNA damage response |
| Homo sapiens | LIN7B | Q9HAP6 | GO:0006887 | exocytosis |
| Homo sapiens | KCNA7 | Q96RP8 | GO:0001508 | action potential |
| Homo sapiens | EXOC3L2 | Q2M3D2 | GO:0006887 | exocytosis |
| Homo sapiens | RUFY3 | Q7L099 | GO:0030154 | cell differentiation |
| Homo sapiens | PPP1R12B | O60237 | GO:0007165 | signal transduction |
| Homo sapiens | VAX1 | Q5SQQ9 | GO:0007411 | axon guidance |
| Homo sapiens | MED25 | Q71SY5 | GO:0000122 | negative regulation of transcription by RNA polymerase II |
| Homo sapiens | COPZ2 | Q9P299 | GO:0006886 | intracellular protein transport |
| Homo sapiens | RBMS1 | P29558 | GO:0006396 | RNA processing |
| Homo sapiens | FZD8 | Q9H461 | GO:0001525 | angiogenesis |
| Homo sapiens | NOTUM | Q6P988 | GO:0016055 | Wnt signaling pathway |
| Homo sapiens | ANAPC2 | Q9UJX6 | GO:0030154 | cell differentiation |
| Homo sapiens | EML2 | O95834 | GO:0007605 | sensory perception of sound |
| Homo sapiens | EML2 | O95834 | GO:0007601 | visual perception |
| Homo sapiens | MEN1 | O00255 | GO:0006974 | DNA damage response |
| Homo sapiens | TMEM161A | Q9NX61 | GO:0034644 | cellular response to UV |
| Homo sapiens | GSE1 | Q14687 | GO:0001890 | placenta development |
| Homo sapiens | MAPKAPK2 | P49137 | GO:0006974 | DNA damage response |
| Homo sapiens | PLCD3 | Q8N3E9 | GO:0001525 | angiogenesis |
| Homo sapiens | MAP3K11 | Q16584 | GO:0000165 | MAPK cascade |
| Homo sapiens | SLC25A26 | Q70HW3 | GO:0006811 | monoatomic ion transport |
| Homo sapiens | MYPOP | Q86VE0 | GO:0000122 | negative regulation of transcription by RNA polymerase II |
| Homo sapiens | XKR6 | Q5GH73 | GO:0043652 | engulfment of apoptotic cell |
| Homo sapiens | ANXA7 | P20073 | GO:0010629 | negative regulation of gene expression |
| Homo sapiens | TLE2 | Q04725 | GO:0016055 | Wnt signaling pathway |
| Homo sapiens | POU3F1 | Q03052 | GO:0045893 | positive regulation of DNA-templated transcription |
| Homo sapiens | ETV2 | O00321 | GO:0016055 | Wnt signaling pathway |
| Homo sapiens | DVL1 | O14640 | GO:0060071 | Wnt signaling pathway |
| Homo sapiens | FCHO2 | Q0JRZ9 | GO:0010324 | membrane invagination |
| Homo sapiens | CFD | P00746 | GO:0006508 | proteolysis |
| Homo sapiens | PLD5 | Q8N7P1 | NOTFOUND | Signaling receptor binding |

**Supplementary Fig 1** Gene ontology (GO) enrichment analysis. (a) Heatmap of top 13 functional enrichment categories analyzed using Metascape of all adaptive genes, colors vary according to the p-value.

**Supplementary Fig 2.** Boxplots show the number of shared PSGs between pairs of samples grouped by (A) phylogenetic relatedness (distant vs close), (B) ecological similarity (different basin vs same basin), and (C) geographic region (different continent vs same continent). Each point represents a pairwise comparison. Boxes indicate the interquartile range (IQR), the horizontal line within boxes represents the median, and whiskers extend to the range of the data.

**Supplementary Fig 3**. Dot plots showing raw RER values (p ≤ 0.01) for each gene and species. Gene-specific averages are highlighted and connected by grey lines for each species group (freshwater and marine).

**Supplementary Fig 4.** Heatmap showing significantly enriched Gene Ontology (GO) Biological Process terms associated with the set of PSGs detected under the branch-site model implemented in codeml. Rows correspond to enriched GO terms and columns to freshwater cetacean species (Inia geoffrensis, Lipotes vexillifer, Platanista minor, Platanista gangetica, Sotalia fluviatilis, and Neophocaena asiaeorientalis). Color intensity represents the significance of enrichment (−log10 adjusted p-value), with darker colors indicating stronger enrichment. Only GO terms remaining significant after false discovery rate correction (Benjamini–Hochberg, adjusted p < 0.05) are shown.
